# Inhibition of PLK4 might enhance the anti‐tumour effect of bortezomib on glioblastoma via PTEN/PI3K/AKT/mTOR signalling pathway

**DOI:** 10.1111/jcmm.14996

**Published:** 2020-03-03

**Authors:** Jing Wang, Dengpeng Ren, Yan Sun, Chao Xu, Chunhong Wang, Rui Cheng, Lina Wang, Guijun Jia, Jinrui Ren, Jiuhong Ma, Yue Tu, Hongming Ji

**Affiliations:** ^1^ Department of neurosurgery Shanxi academy of medical science Shanxi Bethune Hospital Taiyuan China; ^2^ Department of neurosurgery Central Hospital of Yuncheng city Yuncheng China; ^3^ Neurological intensive care unit Special medical center of PAP Tianjin China; ^4^ Department of neurosurgery Shanxi people's hospital Taiyuan China

**Keywords:** *bortezomib*, *glioblastoma*, *PLK4*, *PTEN/PI3K/AKT/mTOR signalling pathway*

## Abstract

Glioblastoma (GBM) is one of the most common aggressive cancers of the central nervous system in adults with a high mortality rate. Bortezomib is a boronic acid–based potent proteasome inhibitor that has been actively studied for its anti‐tumour effects through inhibition of the proteasome. The proteasome is a key component of the ubiquitin‐proteasome pathway that is critical for protein homeostasis, regulation of cellular growth, and apoptosis. Overexpression of polo‐like kinase 4 (PLK4) is commonly reported in tumour cells and increases their invasive and metastatic abilities. In this study, we established a cell model of PLK4 knockdown and overexpression in LN‐18, A172 and LN‐229 cells and found that knockdown of PLK4 expression enhanced the anti‐tumour effect of bortezomib. We further found that this effect may be mediated by the PTEN/PI3K/AKT/mTOR signalling pathway and that the apoptotic and oxidative stress processes were activated, while the expression of matrix metalloproteinases (MMPs) was down‐regulated. Similar phenomenon was observed using in vitro experiments. Thus, we speculate that PLK4 inhibition may be a new therapeutic strategy for GBM.

## 1 INTRODUCTION

Glioblastoma (GBM) is one of the most common aggressive cancers that occurs in the adult brain, and the survival period after initial diagnosis is only 12‐15 months.^1^ GBM accounts for nearly 70% of all adult brain tumours.^2^ Effective treatment for GBM, which is characterized by rapid proliferation ability and highly infiltrative growth, is still lacking.^3^ Bortezomib is a selective and reversible proteasome inhibitor that was first developed for the treatment of inflammation and cachexia in the late 90 seconds.^4^ Studies using several tumour cells lines from the National Cancer Institute (NCI) confirmed its potency and wide range of anti‐tumour effects.^5^ Bortezomib was initially approved by US Food and Drug Administration (FDA) for treatment of refractory multiple myeloma (MM). Previous studies have shown that bortezomib disrupts multiple downstream signalling pathways, such as the NF‐κB signalling pathway and ubiquitin‐proteasome pathway, and has an important role in the regulation of cell cycle, mitosis, cell viability, proliferation and apoptosis in glioblastoma cells.^6^, ^7^, ^8^ It also blocks autophagy flux mediated by the 26S proteasome, thereby inhibiting the elimination of damaged organelles,^9^ and inhibits the proliferation of glioblastoma. Polo‐like kinase 4 (PLK4) is critical for embryonic development and cell cycle control. PLK4 localizes to the nucleolus during the G_2_ phase, moves into the centrosomes during early M phase, and then into the cleavage furrow during cytokinesis.^10^ The expression of PLK4 remains low in G_0_, and in early‐to‐mid‐G_1_ phase, while it increases in late G_1_, S and G_2_ phases and is at the highest level during mitosis.^11^ A previous study reported that PLK4 knockdown reduces invasion, induces epithelial phenotype in breast cancer cells and plays an important role in cancer metastasis.^12^ Another study found that increase in PLK4 expression enhances the resistance of GBM cells to radiotherapy, and inhibition of PLK4 enhances the effect of temozolomide via reduction of IKBKE phosphorylation; besides, depletion of PLK4 in glioma cells using CRISPR/CAS9 significantly decreased the proliferation, viability and survival of glioma cells, increasing the susceptibility of cancer cells to DNA damage agents.^13^, ^14^ Another study reported that a combination of bortezomib and PLK inhibitor showed a significantly higher anti‐tumour effect compared with bortezomib treatment alone in multiple types of head and neck cancers.^15^ Therefore, we hypothesized that inhibition of PLK4 might enhance the therapeutic effect of bortezomib in the treatment of GBM. In this study, we found that bortezomib could significantly inhibit the proliferation of glioma cells using MTT assay and further found that this effect was mediated by the PTEN/PI3K/AKT/mTOR signalling pathway. We also found that the apoptotic pathway and oxidative stress response were activated following bortezomib treatment, while the degradation of extracellular matrix (ECM) was decreased. Furthermore, inhibition of PLK4 enhanced the effect of bortezomib in glioma cells, while overexpression of PLK4 reduced this effect. Using in vitro experiments, we found similar phenomena as in the xenograft experiments. Thus, we speculate that inhibition of PLK4 expression may be a new therapeutic strategy for GBM.

## 2 MATERIALS AND METHODS

### 2.1 Materials

H‐DMEM (10569044) and FBS (10099141C) were purchased from Gibco. Lipofectamine 3000 Transfection Reagent (L3000150) was purchased from Thermo. CFI‐400945 (S7552) was purchased from Selleck. Bortezomib (5043140001) was purchased from Sigma. FastDigest BsmBI (FD0454) was purchased from Fermentas. T4 PNK (M0201S), Quick Ligase (M2200S), BamHI (R0136S) and XhoI (R0146S) were purchased from NEB. Hifair^TM^ III One Step RT‐qPCR Probe Kit (11145ES50), Hifair III One Step RT‐qPCR SYBR Green Kit (11143ES50) and TRIeasy Total RNA Extraction Reagent (10606ES60) were purchased from Yeasen. Anti‐PLK4 (ab137398), PTEN (ab32199), ATM (ab78), p‐ATM (ab81292), ATR (ab2905), p‐ATR (ab227851), AKT (ab179463), p‐AKT (ab38449), p‐mTOR (ab109268), mTOR (ab2732), HIF‐1α (ab1), MMP2 (ab37150), MMP9 (ab73734), Bcl‐2 (ab692), Bax (ab32503), Superoxide Dismutase 1 (ab13498), Thioredoxin (ab26320), caspase‐3 (ab13847) and cleaved caspase‐3 (ab2302) antibodies were purchased from Abcam. FGF (ab99979), EGF (ab217772), TGF‐β (ab100647) and VEGF (ab222510). ELISA kits were purchased from Abcam. ROS detection kit (E004‐1‐1) was purchased from Nanjing Jiancheng Bioengineering Institute. DNA Damage Quantification Colorimetric Kit (K253‐25) was purchased from Biovision. 293T (CRL‐11268), LN‐18 (CRL‐2610), A172 (CRL‐1620) and LN‐229(CRL‐2611) cells were purchased from American Type Culture Collection (ATCC).

### 2.2 Construction of vector

Full length of PLK4 DNA was obtained by polymerase chain reaction (PCR) method using the following primers: Forward: 5′‐CACTGAATTCCATGGCGACCTGCATCGGGG‐3′, Reverse: 5′‐CTGCGGTACCTTATCAATGAAAATTAGGAG‐3′. The PCR product and pCDNA3.1 were digested with BamHI and XhoI to construct the overexpression vector pCDNA3.1‐PLK4. The PLK4 knockdown vector was constructed as described in a previous study^16^ using the following primers: Forward: 5′‐CACCGATTTACCTTCCTATGATTAT‐3′, Reverse: 5′‐AACTAAATGGAAGGATACTAATA‐3′. Briefly, the vector was first digested with FastDigest BsmBI at 37°C for 30 minutes. The primers were phosphorylated and annealed using T4 PNK by incubating at 37°C for 30 minutes and 95°C for 5 minutes. Then, the digested vector and annealed primers were incubated with Quick Ligase at room temperature for 10 minutes. PLK4 knockdown lentivirus was constructed using 293T cells, and the PLK4 knockdown and overexpression vectors were transfected into A172, LN‐18 and LN‐229 cells using Lipo3000 transfect reagent (Thermo), and stably transfected cells were selected using 800 μg/mL G418.

### 2.3 Cell culture and treatment groups

Cells were divided into four groups: control group (NC), bortezomib treatment group (TG), bortezomib treatment combined with PLK4 inhibition group (PI) and bortezomib treatment combined with PLK4 overexpression group (PO). In the bortezomib treatment group, the cells were treated with bortezomib at a concentration of 500 nmol/L for 24 hours.^17^ The cells were cultured in H‐DMEM medium supplied with 10% FBS, NaHCO_3_ (1.5 g/L) and sodium pyruvate (0.11 g/L) at 37°C in 5% CO_2_. The cells were washed with sterile PBS thrice before performing the subsequent experiments.

### 2.4 Ethical statement

Animal studies were performed according to the principles and procedures outlined in the National Institutes of Health Guide for the Care and Use of Animals, and the experiments in this study were approved by the Health Animal Care and Use Committee of the Shanxi Dayi hospital.

### 2.5 Xenograft experiment using nude mice

Mice were purchased from the PLA academy of military medical sciences. A total of 1.5 × 10^6^ LN‐299 cells were injected subcutaneously in the right lower dorsal side of 28‐week‐old female nude mice. The mice were housed for 20 days to allow tumour cells to engraft, following which the mice were divided into four groups: control group (NC), bortezomib treatment group (TG), bortezomib treatment combined with PLK4 inhibition group (PI, treated with 9.4 mg/kg CFI‐400945), and bortezomib treatment combined with PLK4 adenovirus group (PO). Following treatment for 20 days, serum samples of nude mice and xenograft were collected for ELISA, qPCR and Western blotting.

### 2.6 MTT assay

Cells were seeded into a 96‐well plate and cultured for 24 hours, followed by further incubation with 500 nmol/L bortezomib for 24 hours. MTT assay was performed accordingly as described in a previous study.^18^ Cells were incubated with 5 mg/mL MTT reagent for 5 hours, and the optical density at 560 nm was measured using a CMax Plus microplate reader (MD). The number of cells was measured using a cell counter (Countess, Thermo) each day. A proliferation curve was constructed based on the cell counts.

### 2.7 RNA extraction

RNA extraction was performed according to the following protocol. Briefly, cells were lysed with lysis buffer and incubated at room temperature for 5 minutes. After incubation, the cell lysate was centrifuged at 12 000 g for 10 minutes (4°C). Then, chloroform was added into the cell lysate and oscillated for 15 seconds. The cell lysate was further centrifuged at 12 000 g for 10 minutes (4°C) and washed with 75% ethanol. The RNA was collected by centrifugation at 7500 g for 10 minutes (4°C) and stored at −80°C until it was used in experiments.

### 2.8 Reverse transcription and real‐time quantitative polymerase chain reaction (qPCR)

Reverse transcription was performed according to the following protocol. Briefly, the reaction mixture was made up as recommended and the reaction steps were set up as follows: reverse transcription: 50°C for 15 minutes; pre‐degeneration: 95°C for 30 seconds; and amplification reaction: 95°C for 10 seconds, 60°C for 30 seconds, and repeated for 45 cycles. Then, qPCR was performed according to the protocol, and the reaction steps were set up as follows: reverse transcription: 42°C for 10 minutes; pre‐degeneration: 95°C for 5 minutes; and amplification reaction: 95°C for 10 seconds, 60°C for 30 seconds, and repeated for 40 cycles. The primers used were as follows: TNF‐α: Forward: 5′‐CGAGTGACAAGCCTGTAGC‐3′, Reverse: 5′‐GGTGTGGGTGAGGAGCACAT‐3′; Caspase‐9: Forward: 5′‐GGCTGTCTACGGCACAGATGCA‐3′, Reverse: 5′‐CTGGCTCGGGGTTACTGCCAG‐3′; Caspase‐3: Forward: 5′‐TTGAGACAGACAGTGGTGTTGATGATG‐3′, Reverse: 5′‐ATAATAACCAGGTGCTGTGGAGTATGC‐3′; Bax: Forward: 5′‐CCCGAGAGGTCTTCTTCCG‐3′, Reverse: 5′‐GAAGTCCAGTGTCCAGCCCA‐3′. Relative gene expression was determined using the 2‐ΔΔCq method.^19^

### 2.9 Western blotting

Cells in each group were lysed with lysis buffer (8M Urea, 50 mmol/L IAA, 10 mmol/L DTT and supplemented with proteinase inhibitor cocktail). Following ultrasonic lysis, proteins in the supernatant were collected by centrifugation at 12 000 rpm for 10 minutes. The concentrations of the protein samples were determined using the BCA assay, and then, the protein samples were separated using SDS‐PAGE. Briefly, 60 μg of proteins per sample was separated using 10% SDS‐PAGE, and after electrophoresis, the proteins were transferred onto a 0.22 μm nitrocellulose membrane using a semi‐dry criterion. The membranes were incubated with 5% skim milk at room temperature for 1 hours, followed by incubation with primary antibodies overnight at 4°C. Then, the membranes were incubated with the secondary antibody at room temperature for 1 hours. The grey value of each group was detected using chemiluminescence and analysed using the IPP 6.0 software.

### 2.10 Flow cytometry

Cells were first grouped and treated as previously described. The cells in each group were fixed in 70% ethanol overnight followed by incubation with 20 μg/mL RNase A for 30 minutes at 37°C. After washing with PBS, the cells were incubated with 50 μg/mL at room temperature for 30 minutes away from light. The apoptosis cells were detected using FC500 flow cytometer (Beckman).

### 2.11 Detection of cellular ROS concentration

Detection of cellular ROS concentration was performed according to the following protocol. Briefly, the cells and xenograft tissues were digested with trypsin and then washed with PBS three times. The cells were then incubated with the detection probe at a concentration of 10 μmol/L for 30 minutes. After resuspending in PBS, the fluorescence intensity was detected using a Multiskan FC microplate reader.

### 2.12 Measurement of DNA damage

Cellular DNA was extracted using a DNA extraction kit (D1700, Solarbio). Briefly, the cells were incubated with Buffer A and RNase A for 15 minutes at 55°C followed by incubation with proteinase K for 3 hours at 55°C. Then, the reaction mixture was incubated with buffer B for 30 minutes at 75°C, and the samples were passed through the absorption tube. After washing with the wash buffer, the DNA was eluted using the elution buffer. The concentration of DNA was determined using a Nanodrop 3300 Fluorospectrometer (Thermo). DNA samples were diluted to 0.1 μg/μL to quantitate the DNA damage. A DNA damage quantification colorimetric kit (K253‐25) was used to detect DNA damage. DNA samples were incubated with the ARP solution for 1 hours at 37°C and then incubated further at −20°C for 1 minutes after mixing with TE, glycogen and ethanol. After centrifugation at high speed for 10 minutes, the pellets were washed with 70% ethanol. The DNA samples were added into each well of a 96‐well plate, washed with wash buffer and incubated with HRP developer at 37°C for 1 hour. The optical density was measured at 650 nm using a Multiskan FC microplate reader (Thermo).

### 2.13 Statistical analysis

The data are presented as the mean ± SEM Each experiment was repeated three times independently. One‐way ANOVA was used to analyse the differences between groups using GraphPad 7.0 software. *P* value <.05 was set as a statistically significant difference.

## 3 RESULTS

### 3.1 Effect of bortezomib on proliferation of glioma cells

**Figure 1:**
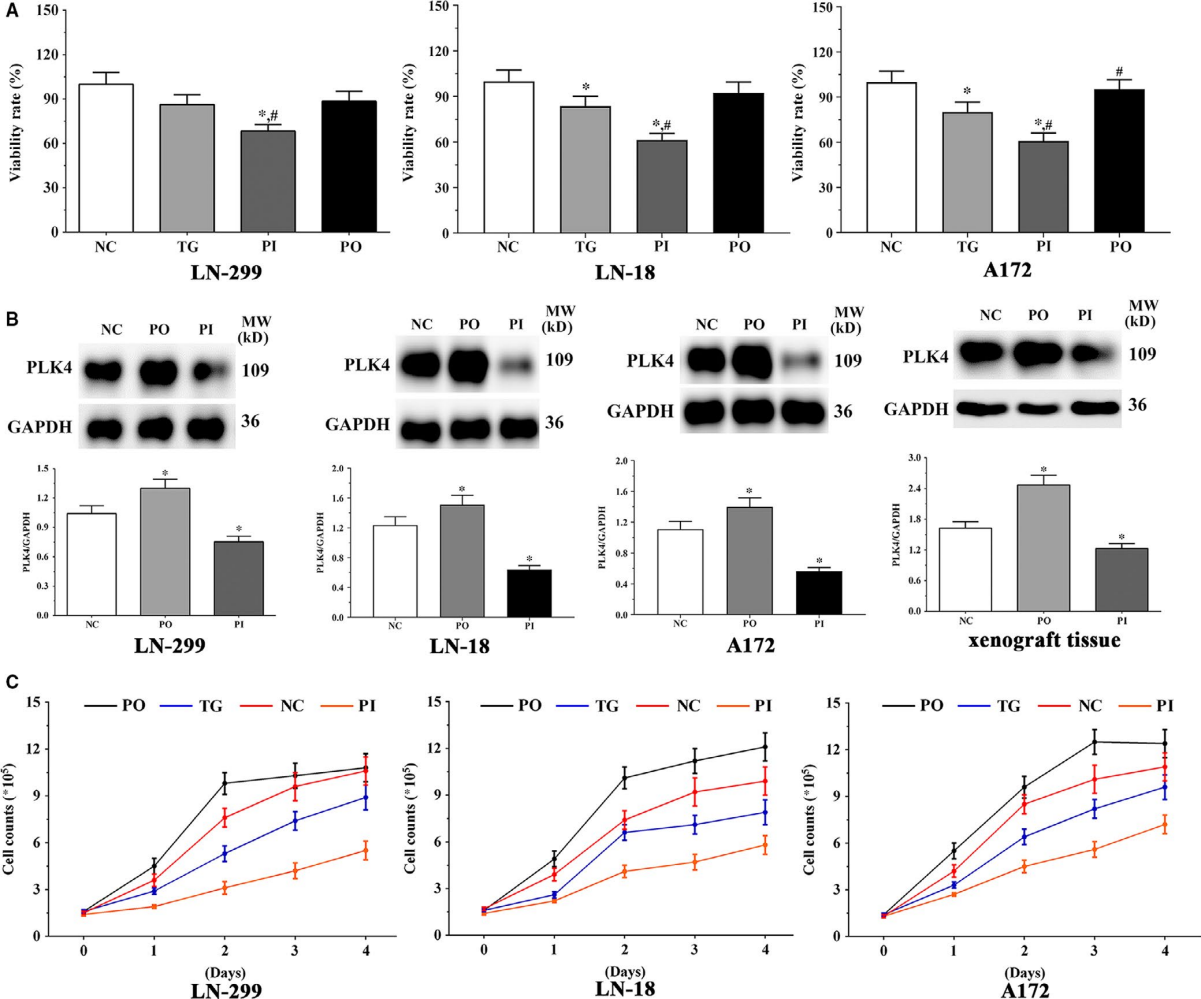
Effect of bortezomib on the proliferation of cells. A, Viability of LN‐229, LN‐18 and A172 cells in each group. B, The expression of PLK4 in LN‐299, LN‐18, and A172 cells and xenograft tissues. C, Proliferation curve of LN‐229, LN‐18 and A172 cells in each group. Data were presented as mean ± SD (n = 3). NC, control group, TG, bortezomib treatment group, PI, bortezomib treatment combined with PLK4 inhibition group, PO, bortezomib treatment combined with PLK4 overexpression group. *P < .05 vs NC group; #*P* < .05 vs TG group

As shown in Figure 1A, the viability of LN‐229 cells decreased significantly in the PI group (*P* < .05) compared to the NC and TG groups; however, the viability in PO group did not change significantly (*P* > .05). The expression of PLK4 was significantly higher in the PO group (*P* < .05), and significantly lower in the PI group (*P* < .05) compared to the NC group (Figure 1B). The expression of PLK4 in the xenografts was significantly higher in the PO group (*P* < .05) and lower in the PI group (*P* < .05).

### 3.2 Effect of bortezomib on expression of apoptosis‐related genes in glioma cells

**Figure 2:**
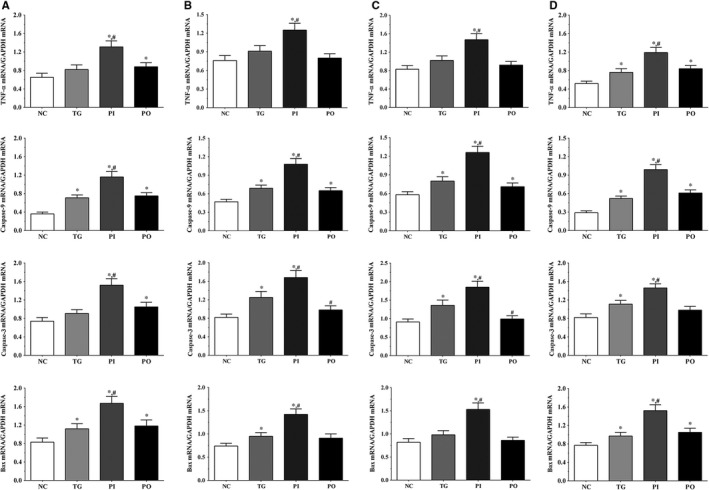
Effect of bortezomib on expression of pro‐inflammatory cytokines. A Expression of TNF‐α, caspase‐9, caspase‐3, and Bax in LN‐229 cells. B Expression of TNF‐α, caspase‐9, caspase‐3 and Bax in LN‐18 cells. C, Expression of TNF‐α, caspase‐9, caspase‐3 and Bax in A172 cells. D, Expression of TNF‐α, caspase‐9, caspase‐3 and Bax in xenograft tissues. Data were presented as mean ± SD (n = 3). NC, control group, TG, bortezomib treatment group, PI, bortezomib treatment combined with PLK4 inhibition group, PO, bortezomib treatment combined with PLK4 overexpression group. *P < .05 vs NC group; #*P* < .05 vs TG group

As shown in Figure 2, the expression of apoptosis‐related genes in the glioma cells was measured using qPCR method. In the LN‐229 cells, compared to the NC group, the expression of TNF‐α was significantly higher in the PI and PO groups (*P* < .05), and compared to the TG group, the expression was significantly higher in the PI group (*P* < .05). Compared to the NC group, the expression of caspase‐9 was significantly higher in all treatment groups (*P* < .05), and compared to the TG group, the expression was significantly higher in the PI group (*P* < .05). The expression of caspase‐3 was significantly increased in the PI and PO groups (*P* < .05), and compared to the TG group, the expression was significantly increased in the PI group (*P* < .05). The expression of Bax was significantly increased in all treatment groups compared to the NC group (*P* < .05), and compared to the TG group, the expression was significantly increased in the PI group (*P* < .05). Similar trends were observed in the LN‐18 and A172 cells. In serum samples, the expression of TNF‐α was significantly increased in all treatment groups compared to the NC group (*P* < .05), and compared to the TG group, the expression of TNF‐α was significantly increased in the PI group (*P* < .05). The change in expression of caspase‐9 presented a similar trend to that of TNF‐α. The expression of caspase‐3 was significantly increased in the TG and PI group compared to the NC group (*P* < .05), and compared to the TG group, the expression of caspase‐3 was significantly increased in the PI group (*P* < .05). The expression of Bax was significantly increased in all treatment groups compared to the NC group (*P* < .05), and compared to the TG group, the expression of Bax was significantly increased in the PI group (*P* < .05).

### 3.3 Detection of apoptotic cells by flow cytometry

**Figure 3:**
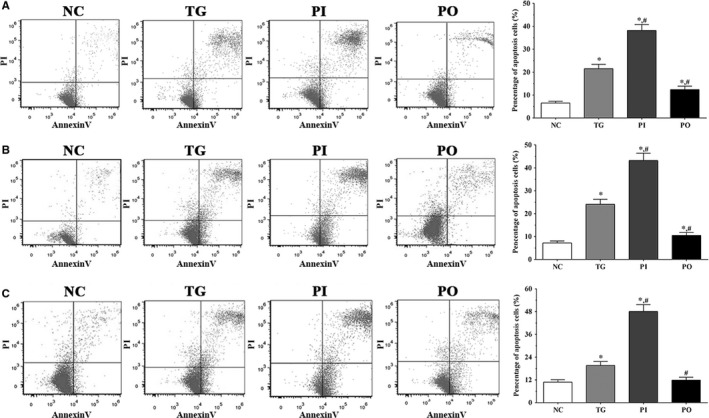
Detection of apoptotic cells of LN‐299 (A), LN‐18 (B) and A172 (C) cells using flow cytometry. NC, control group, TG, bortezomib treatment group, PI, bortezomib treatment combined with PLK4 inhibition group, PO, bortezomib treatment combined with PLK4 overexpression group

As shown in Figure 3, apoptotic cells were detected using flow cytometry. In LN‐299, LN‐18 and A172 cells, the proportion of apoptotic cells were significantly higher in the TG and PI groups (*P* < .05) compared to the NC group, and significantly higher in the PI group compared to the TG group (*P* < .05). However, these changes were not significant following overexpression of PLK4.

### 3.4 Effect of bortezomib on expression of caspase‐3 and caspase‐9

**Figure 4:**
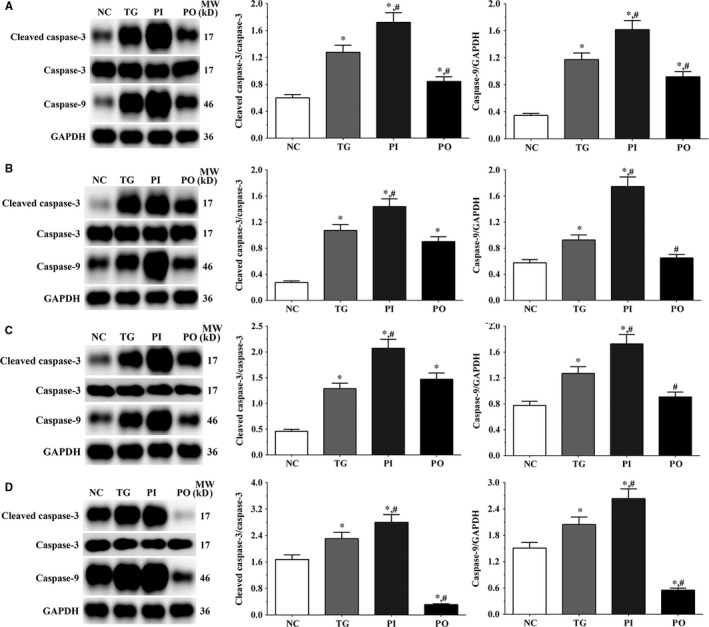
Expression of caspase‐3 and caspase‐9 in each group. A, Expression of caspase‐3 and caspase‐9 in each group of LN‐299 cells. B, Expression of caspase‐3 and caspase‐9 in each group of LN‐18 cells. C, Expression of caspase‐3 and caspase‐9 in each group of A172 cells. (D) Expression of caspase‐3 and caspase‐9 in each group of xenografts tissues. Data were presented as mean ± SD (n = 3). NC, control group, TG, bortezomib treatment group, PI, bortezomib treatment combined with PLK4 inhibition group, PO, bortezomib treatment combined with PLK4 overexpression group. *P < .05 vs NC group; #*P* < .05 vs TG group

As shown in Figure 4, the ratio of cleaved caspase‐3/caspase‐3 in LN‐299, LN‐18, A172 cells and xenografts was significantly increased in TG and PI groups (*P* < .05) compared with NC group, and the ratio was decreased in PO group (*P* < .05) compared with NC group. And compared with TG group, the ratio of cleaved caspase‐3/caspase‐3 was significantly increased in PI group (*P* < .05) and decreased in PO group. The changing in expression of caspase‐9 presented a similar trend with the ratio of cleaved caspase‐3/caspase‐3.

### 3.5 Effect of bortezomib on activation of PTEN/PI3K/AKT/mTOR signalling pathway in glioma cells

**Figure 5:**
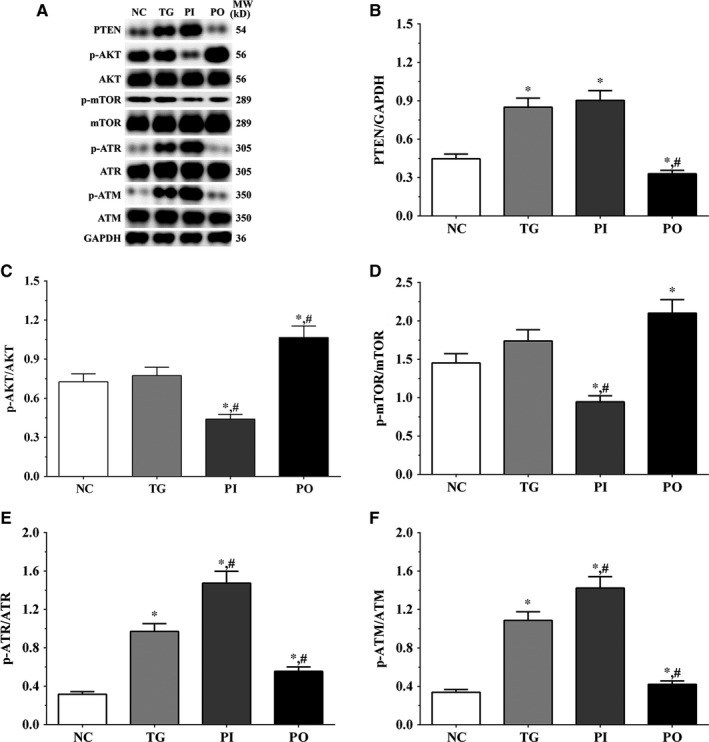
Effect of bortezomib on activation of PTEN/PI3K/AKT/mTOR signalling pathway in LN‐299 cells. A, Expression of PTEN, p‐AKT, AKT, p‐mTOR, mTOR, p‐ATR, ATR, p‐ATM and ATM using Western blotting. B, C, D, E and F, Quantitative analysis of each protein. Data were presented as mean ± SD (n = 3). NC, control group, TG, bortezomib treatment group, PI, bortezomib treatment combined with PLK4 inhibition group, PO, bortezomib treatment combined with PLK4 overexpression group. *P < .05 vs NC group; #*P* < .05 vs TG group

**Figure 6:**
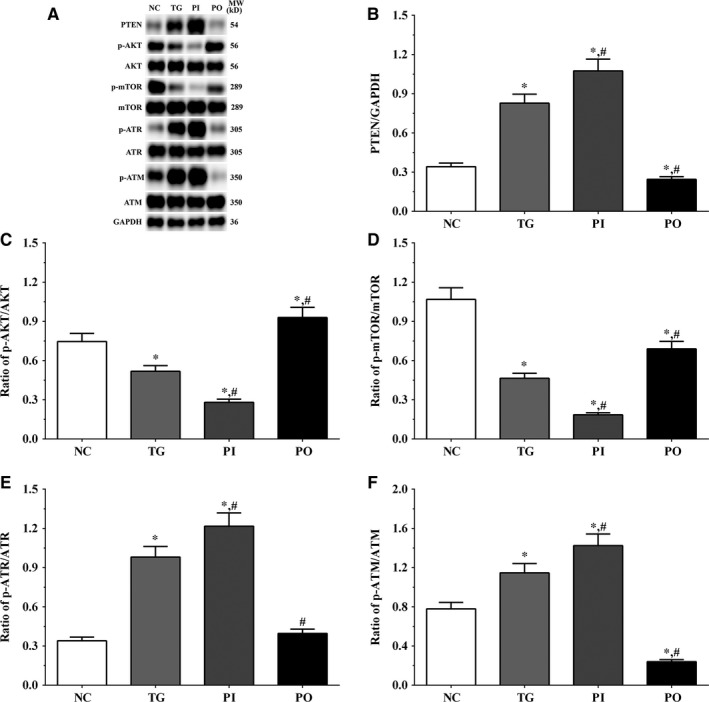
Effect of bortezomib on activation of PTEN/PI3K/AKT/mTOR signalling pathway in LN‐18 cells. A, Expression of PTEN, p‐AKT, AKT, p‐mTOR, mTOR, p‐ATR, ATR, p‐ATM and ATM using Western blotting. B, C, D, E and F, Quantitative analysis of each protein. Data were presented as mean ± SD (n = 3). NC, control group, TG, bortezomib treatment group, PI, bortezomib treatment combined with PLK4 inhibition group, PO, bortezomib treatment combined with PLK4 overexpression group. *P < .05 vs NC group; #*P* < .05 vs TG group

**Figure 7:**
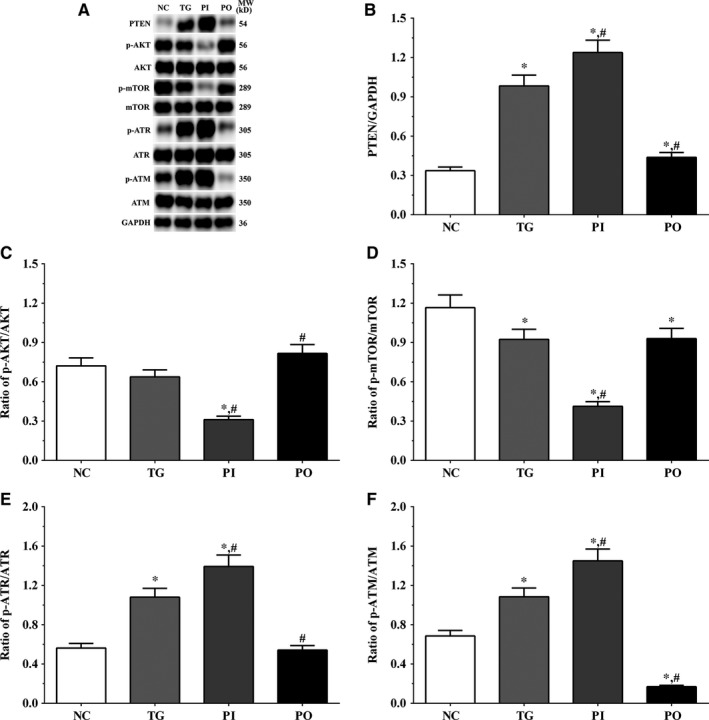
Effect of bortezomib on activation of PTEN/PI3K/AKT/mTOR signalling pathway in A172 cells. A, Expression of PTEN, p‐AKT, AKT, p‐mTOR, mTOR, p‐ATR, ATR, p‐ATM and ATM using Western blotting. B, C, D, E and F, Quantitative analysis of each protein. Data were presented as mean ± SD (n = 3). NC, control group, TG, bortezomib treatment group, PI, bortezomib treatment combined with PLK4 inhibition group, PO, bortezomib treatment combined with PLK4 overexpression group. *P < .05 vs NC group; #*P* < .05 vs TG group

**Figure 8:**
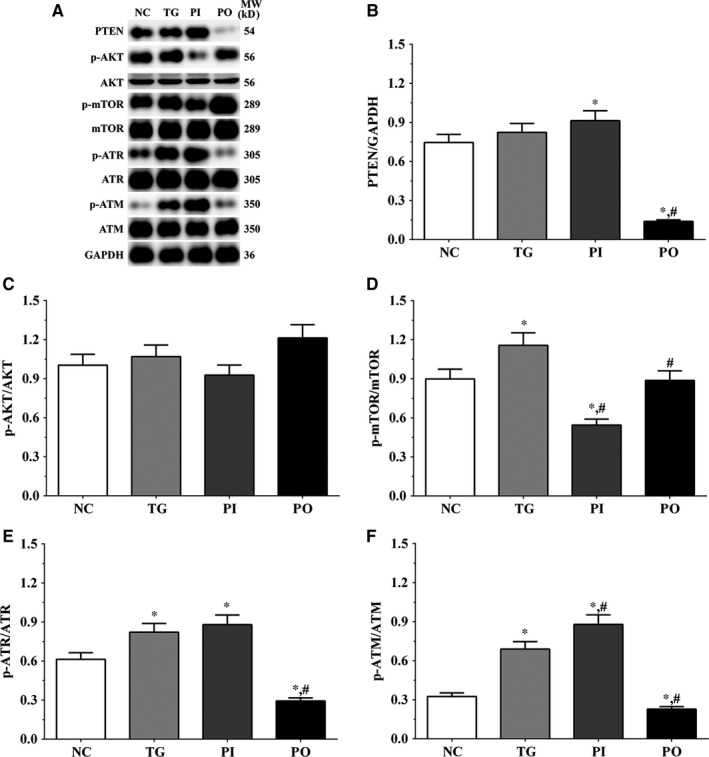
Effect of bortezomib on activation of PTEN/PI3K/AKT/mTOR signalling pathway in xenograft tissues. A, Expression of PTEN, p‐AKT, AKT, p‐mTOR, mTOR, p‐ATR, ATR, p‐ATM and ATM using Western blotting. B, C, D, E and F, Quantitative analysis of each protein. Data were presented as mean ± SD (n = 3). NC, control group, TG, bortezomib treatment group, PI: bortezomib treatment combined with PLK4 inhibition group, PO, bortezomib treatment combined with PLK4 overexpression group. *P < .05 vs NC group; #*P* < .05 vs TG group

As shown in Figure 5, the expression of PTEN was significantly higher in the TG and PI groups (*P* < .05), and significantly lower in the PO group (*P* < .05) compared to the NC group. The expression of PTEN decreased significantly in the PO group (*P* < .05) compared to the TG group. The ratio of p‐AKT/AKT decreased significantly in the PI and increased significantly in PO groups (*P* < .05) compared to the NC and TG groups. The ratio of p‐mTOR/mTOR was significantly lower in the PI group (*P* < .05) and higher in PO group (*P* < .05) compared to the NC group. The ratio of p‐mTOR/mTOR was significantly lower in PI group (*P* < .05) compared to the TG group. The ratio of p‐ATR/ATR was significantly higher in all the treatment groups (*P* < .05) compared to the NC group and was higher in the PI group (*P* < .05) and was significantly lower in the PO group (*P* < .05) compared to the TG group. Changes in the ratio of p‐ATM/ATM presented a similar trend as that of p‐ATR/ATR. Similar trends were also observed in A172 and LN‐18 cells (Figures 6, 7). In the xenografts (Figure 8), the expression of PTEN was significantly higher in the PI group (*P* < .05) and lower in the PO group (*P* < .05) compared to the NC group. The expression of PTEN was significantly lower in the PO group (*P* < .05) compared to the TG group. However, the change in p‐AKT/AKT was lower in the PI group and higher in the PO group although the difference was not significant. The ratio of p‐mTOR/mTOR was significantly higher in the TG group (*P* < .05) and significantly lower in PI group (*P* < .05) compared to the NC group. The ratio of p‐mTOR/mTOR was significantly lower in the PI and PO groups (*P* < .05) compared to the TG group. The ratio of p‐ATR/ATR was significantly higher in the TG and PI groups (*P* < .05) and significantly lower in the PO group (*P* < .05) compared to the NC group. The ratio of p‐ATR/ATR was significantly lower in the PO group (*P* < .05) compared to the TG group. The change in p‐ATM/ATM was significantly higher in the TG and PI groups (*P* < .05) and significantly lower in the PO group (*P* < .05) compared to the NC group. The change in p‐ATM/ATM was significantly higher in the PI group (*P* < .05) and significantly lower in the PO group (*P* < .05) compared to the TG group. However, the change in the p‐mTOR/mTOR ratio in TG group of the xenografts was different from that observed in the cell lines, and we believe that there may be other regulatory pathways in the expression of mTOR, and this remains to be explored further in our future studies.

### 3.6 Effect of bortezomib on the expression of target proteins in glioma cells

**Figure 9:**
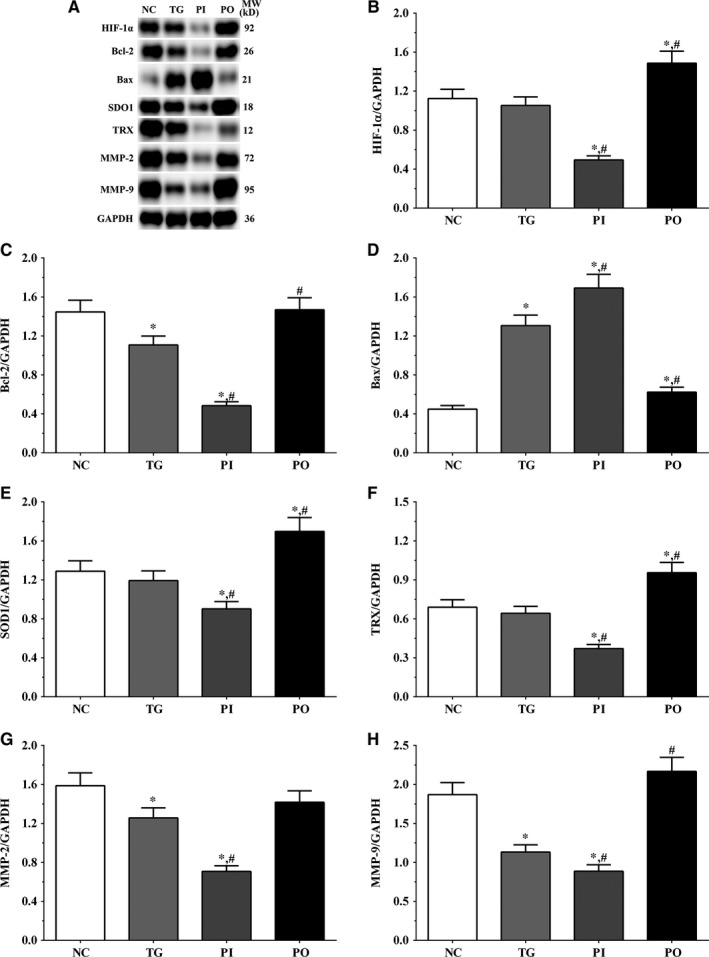
Effect of bortezomib on activation of target proteins in LN‐299 cells. A, Expression of HIF‐1α, Bcl‐2, Bax, SOD1, TRX, MMP‐2 and MMP‐9 using Western blotting. B, C, D, E and F, Quantitative analysis of each protein. Data were presented as mean ± SD (n = 3). NC, control group, TG, bortezomib treatment group, PI, bortezomib treatment combined with PLK4 inhibition group, PO, bortezomib treatment combined with PLK4 overexpression group. *P < .05 vs NC group; #*P* < .05 vs TG group

**Figure 10:**
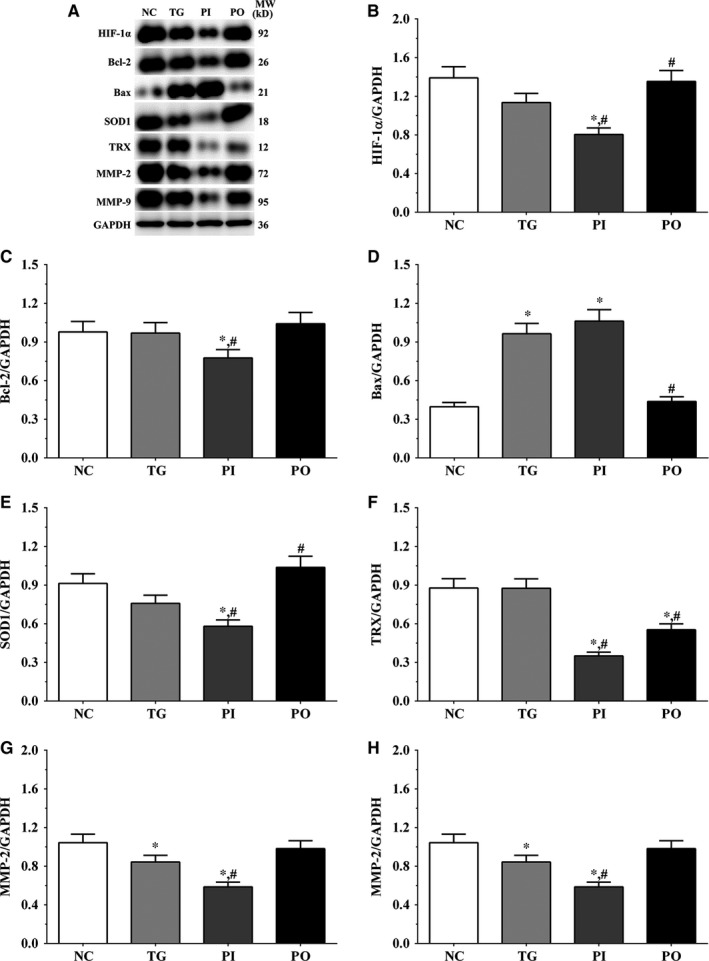
Effect of bortezomib on activation of target proteins in LN‐18 cells. A, Expression of HIF‐1α, Bcl‐2, Bax, SOD1, TRX, MMP‐2 and MMP‐9 using Western blotting. B, C, D, E and F, Quantitative analysis of each protein. Data were presented as mean ± SD (n = 3). NC, control group, TG, bortezomib treatment group, PI, bortezomib treatment combined with PLK4 inhibition group, PO, bortezomib treatment combined with PLK4 overexpression group. *P < .05 vs NC group; #*P* < .05 vs TG group

**Figure 11:**
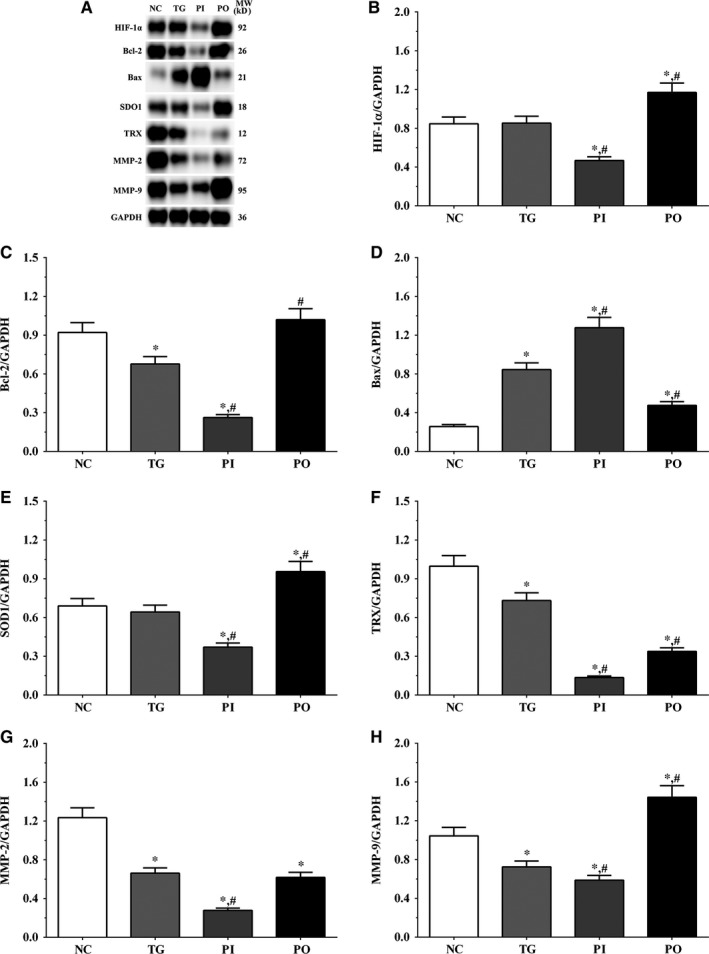
Effect of bortezomib on activation of target proteins in A172 cells. A, Expression of HIF‐1α, Bcl‐2, Bax, SOD1, TRX, MMP‐2 and MMP‐9 using Western blotting. B, C, D, E and F, Quantitative analysis of each protein. Data were presented as mean ± SD (n = 3). NC, control group, TG, bortezomib treatment group, PI, bortezomib treatment combined with PLK4 inhibition group, PO, bortezomib treatment combined with PLK4 overexpression group. *P < .05 vs NC group; #*P* < .05 vs TG group

**Figure 12:**
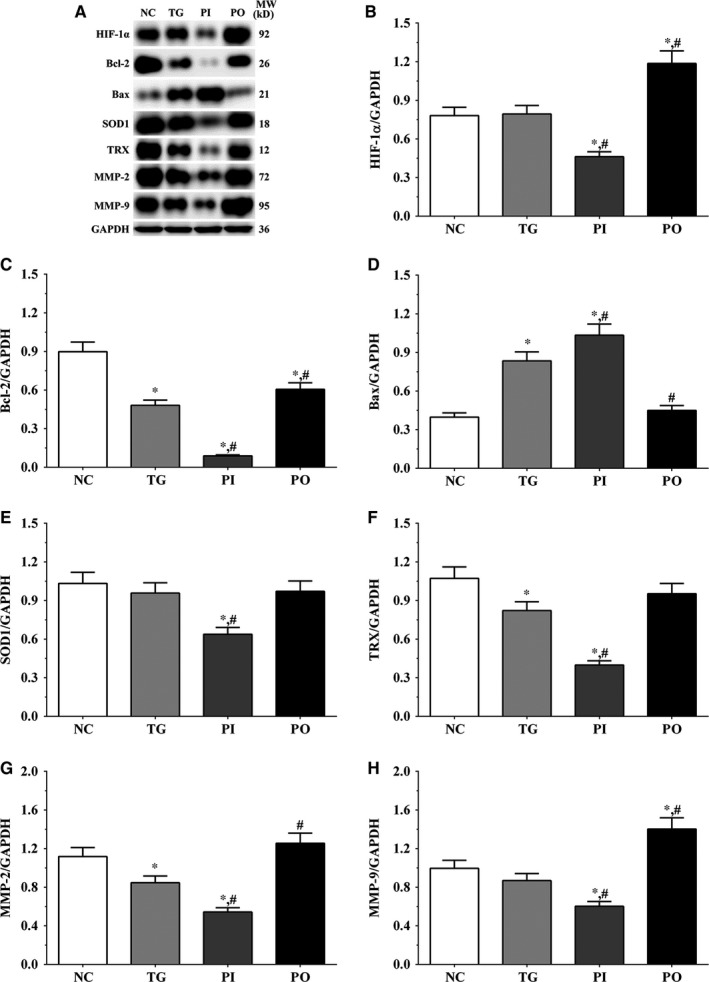
Effect of bortezomib on activation of target proteins in xenograft tissues. A, Expression of HIF‐1α, Bcl‐2, Bax, SOD1, TRX, MMP‐2 and MMP‐9 using Western blotting. B, C, D, E and F, Quantitative analysis of each protein. Data were presented as mean ± SD (n = 3). NC, control group, TG, bortezomib treatment group, PI, bortezomib treatment combined with PLK4 inhibition group, PO, bortezomib treatment combined with PLK4 overexpression group. *P < .05 vs NC group; #*P* < .05 vs TG group

As shown in Figure 9, the expression of HIF‐1α was significantly lower in the PI group (*P* < .05) and significantly higher in the PO group (*P* < .05) compared to the NC and TG groups. The expression of Bcl‐2 decreased significantly in all the treatment groups (*P* < .05) compared to the NC group. The expression of Bcl‐2 decreased significantly in the PI group (*P* < .05) and increased significantly in PO group compared to the TG group. The expression of Bax increased significantly in the TG and PI groups (*P* < .05) compared to the NC group and also increased significantly in the PI group (*P* < .05) while it decreased significantly in the PO group (*P* < .05) compared to the TG group. The expression of SOD1 decreased significantly in the PI group compared to the NC and TG groups (*P* < .05). The expression of TRX was significantly lower in the TG and PI groups (*P* < .05) compared to the NC group and was significantly lower in the PI group (*P* < .05) compared to the TG group. The expression of MMP‐2 decreased significantly in the TG and PI groups (*P* < .05) compared to the NC group, and the expression of MMP‐2 decreased significantly in the PI group (*P* < .05) while it increased significantly in the PO group (*P* < .05), compared to the TG group. The expression of MMP‐9 decreased significantly in the PI group (*P* < .05) and increased significantly in the PO group (*P* < .05) compared to the NC and TG groups. Similar trends were observed in the A172 and LN‐18 cells (Figures 10, 11). In the xenografts (Figure 12), the expression of HIF‐1α decreased significantly in the PI group (*P* < .05) compared to the NC and TG groups and increased significantly in the PO group (*P* < .05) compared to the TG group. The expression of Bcl‐2 was significantly lower in the PI group (*P* < .05) compared to the NC and TG groups. The expression of Bax was significantly higher in the TG and PI groups (*P* < .05) compared to the NC group, and significantly lower in the PO group (*P* < .05) compared to the TG group. The expression of SOD1 was significantly lower in the PI group (*P* < .05) compared to the NC and TG groups, and significantly lower in the PO group (*P* < .05) compared to the TG group. The expression of TRX was significantly lower in the PI and PO groups (*P* < .05) compared to the NC and TG groups. The expression of TRX increased in the PO group compared to the PI group although the change was not significant. The expression of MMP‐2 decreased significantly in TG and PI groups (*P* < .05) compared to the NC group and also decreased significantly in the PI group (*P* < .05) compared to the TG group. The change in expression of MMP‐9 presented a similar trend as that of MMP‐2.

### 3.7 Effect of bortezomib on secretion of angiogenesis‐related molecules in glioma cells

**Figure 13:**
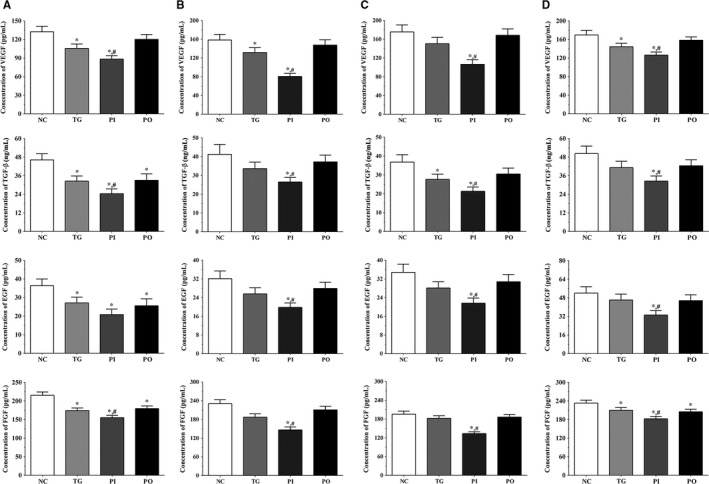
Effect of bortezomib on secretion of growth factor in culture medium and serum sample of nude mice. A, Concentration of VEGF, TGF‐β, EGF and FGF in culture medium of LN‐299 cells. B, Concentration of VEGF, TGF‐β, EGF and FGF in culture medium of LN‐18 cells. C, Concentration of VEGF, TGF‐β, EGF and FGF in culture medium of A172 cells. D, Concentration of VEGF, TGF‐β, EGF and FGF in serum samples of nude mice. Data were presented as mean ± SD (n = 3). NC, control group, TG, bortezomib treatment group, PI, bortezomib treatment combined with PLK4 inhibition group, PO, bortezomib treatment combined with PLK4 overexpression group. *P < .05 vs NC group; #*P* < .05 vs TG group

As shown in Figure 13, the concentration of angiogenesis‐related molecules in the culture medium of the LN‐299 cells was measured using ELISA. In LN‐299 cells, compared with NC group, the concentration of vascular endothelial growth factor (VEGF) was significantly lower in the TG and PI groups (*P* < .05), and the concentration of VEGF in PI group was significantly lower compared to the TG group (*P* < .05). The concentration of transforming growth factor‐β (TGF‐β) was significantly lower in all the treatment groups compared to the NC group (*P* < .05), and compared to the TG group, the concentration of TGF‐β was significantly lower in the PI group (*P* < .05). The concentration of epidermal growth factor (EGF) also decreased significantly in all the treatment groups compared to the NC group (*P* < .05). The concentration of fibroblast growth factor (FGF) was significantly lower in all treatment groups compared to the NC group (*P* < .05), and significantly lower in the PI group compared to the TG group (*P* < .05). Changes in these factors in the LN‐18 and A172 cells presented a similar trend as that in the LN‐299 cells. Compared to the NC group, the concentration of VEGF was significantly lower in the TG and PI groups (*P* < .05), and the concentration of VEGF in PI group was significantly lower compared to the TG group (*P* < .05). The concentration of TGF‐β was significantly lower in the PI group compared to the NC and TG groups (*P* < .05). The concentration of EGF was significantly lower in the PI group compared to the NC and TG groups (*P* < .05). The concentration of FGF was significantly lower in all treatment groups compared to the NC group (*P* < .05) and was significantly lower in the PI group compared to the TG group (*P* < .05).

### 3.8 Relative concentration of cellular ROS

**Figure 14:**
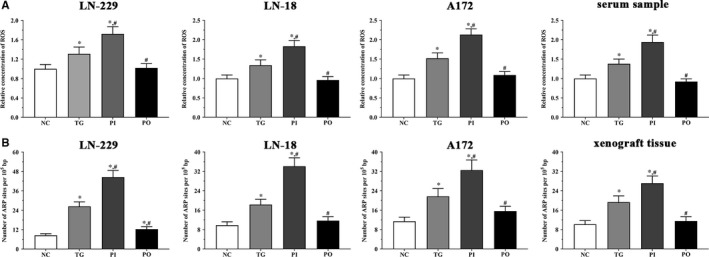
Concentration of ROS and number of ARP site in each group of LN‐299, LN‐18 and A172 cells and nude mice. A, Concentration of ROS in cultured medium of cells and serum samples of nude mice. B, Number of ARP site in cultured medium of cells and xenograft. Data were presented as mean ± SD (n = 3). NC, control group, TG, bortezomib treatment group, PI, bortezomib treatment combined with PLK4 inhibition group, PO, bortezomib treatment combined with PLK4 overexpression group. *P < .05 vs NC group; #*P* < .05 vs TG group

As shown in Figure 14A, relative concentration of cellular ROS in LN‐299 cells was significantly higher in the TG and PI groups (*P* < .05) compared to the NC group, and significantly higher in the PI group (*P* < .05) compared to the TG group. The concentration of cellular ROS was significantly lower in the PO group (*P* < .05) compared to the TG group, although it did not change significantly in the PO group compared to the NC group. Similar trends were observed in the LN‐18 and A172 cells, and in the xenografts*.*

### 3.9 Quantification of DNA damage

As shown in Figure 14B, DNA damage in LN‐299 cells was significantly higher in all treatment groups (*P* < .05) compared to the NC group, and significantly higher in the PI group (*P* < .05) compared to the TG group. DNA damage was significantly lower in the PO group (*P* < .05) compared to the TG group. Similar trends were observed in the LN‐18 and A172 cells, and in the xenograft tissues*.*

### 3.10 Measurement of the diameter of xenograft tissues

**Figure 15:**
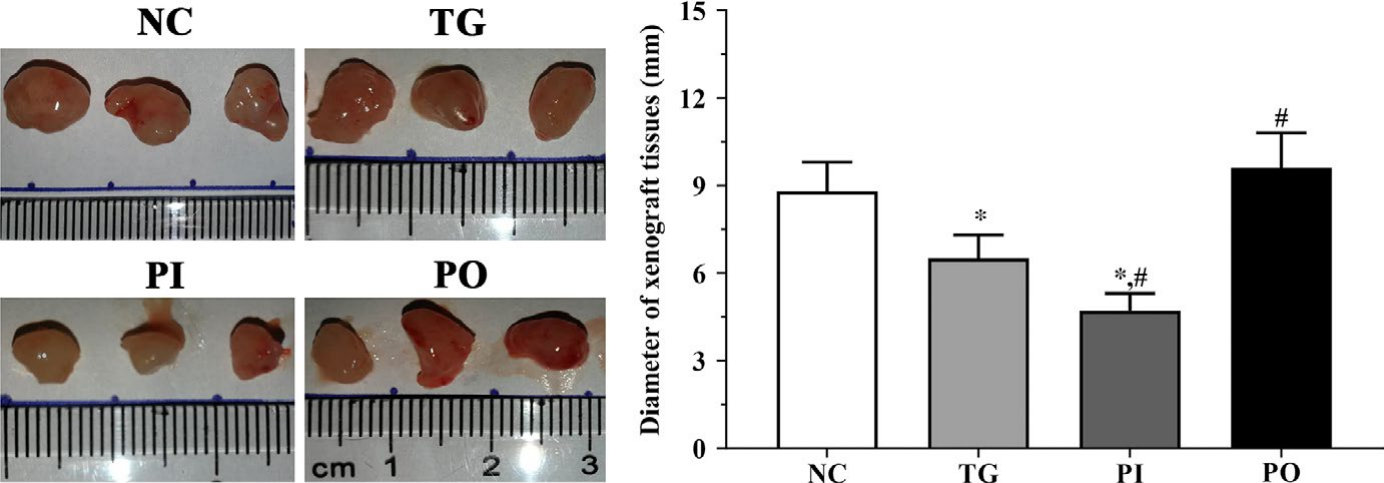
Measurement of the diameter of xenograft tissues in each group. Data were presented as mean ± SD (n = 3). NC, control group, TG, bortezomib treatment group, PI, bortezomib treatment combined with PLK4 inhibition group, PO, bortezomib treatment combined with PLK4 overexpression group. *P < .05 vs NC group; #*P* < .05 vs TG group

As shown in Figure 15, the diameter of xenograft tissues was significantly decreased in TG group and PI group (*P* < .05) compared with NC group and was not significantly changed in PO group. And compared with TG group, the diameter of xenograft tissues was significantly decreased in PI group (*P* < .05) and significantly increased in PO group (*P* < .05).

## 4 DISCUSSION

Gliomas are primary tumours that originate from neuroglial stem or progenitor cells. They constitute nearly 30% of brain and central nervous system tumours, and 80% of all malignant brain tumours.^20^ Gliomas are the most common primary intracranial tumours in adults, and half of all newly diagnosed gliomas are classified as glioblastomas, which are more common than low‐grade gliomas.^21^ The occurrence of glioblastoma is associated with a short survival and fatal outcome with limited treatment options. The PLK family was first discovered over 30 years ago^22^ and consists of PLK1, PLK2, PLK3, PLK4 and PLK5. Among these, PLK4 plays a critical role in the regulation of centriole duplication and mitotic progression.^23^ The dysregulation of PLK4 has been shown to induce the loss of centrosome numeral integrity, which leads to genomic instability and tumorigenesis. In this study, we first established a PLK4 overexpression and knockdown cell model in LN‐229 cells and found that while bortezomib treatment inhibited the proliferation of these cells, knockdown of PLK4 enhanced this effect. We found that this effect may be mediated by the activation of ATM/ATR via the PTEN/PI3K/mTOR signalling pathway. Besides, we also found that the oxidative stress process was activated following bortezomib treatment, while the expression of MMPs and cell cycle‐related molecules was inhibited. These phenomena could be enhanced by knockdown of PLK4, and therefore, inhibition of PLK4 might be an effective therapeutic strategy for glioma. Similar trends were observed using in vitro experiments.

Phosphatase and tensin homologue (PTEN) is a key tumour suppressor, which is also essential for normal cell maintenance.^24^ PTEN interacts with cytoplasmic targets and has a regulatory role in cell proliferation, apoptosis, cell cycle progression, migration and invasion. PTEN also regulates the stability of chromosomal and DNA double‐strand break repair.^25^, ^26^ PTEN is also pivotal in the negative regulation of the PI3K/AKT signalling pathway, where it has a dephosphorylation function in the conversion of PIP2 into PIP3.^27^ Loss of PTEN is commonly observed in many tumours and leads to the activation of PI3K/AKT pathway and multiple downstream molecules including focal adhesion kinase (FAK), insulin receptor substrate 1 and c‐SRC.^28^ mTOR is a downstream molecule in the PI3K/AKT signalling pathway and belongs to the phosphoinositide kinase‐related family of protein kinases (PIKK).^29^ A previous study found that reducing mTOR activity increases HIF‐1α activity and the production of reactive oxygen species (ROS), leading to the oxidative stress in cells.^30^ Reduction in mTOR activity leads to the phosphorylation of 4E‐BP1, resulting in dissociation with the cap‐binding protein eukaryotic initiation factor 4E (eIF4E), and reduction of cap‐dependent mRNA translation.^31^ In this study, we found that following bortezomib treatment, the activation of PI3K/mTOR signalling pathway was inhibited, with increased expression of PTEN. We also noticed that the activation of ATM/ATR was increased, and inhibition of PLK4 enhanced these effects. Hence, we speculate that bortezomib could significantly decrease the activation of the PI3K/mTOR signalling pathway and increase the expression of PTEN, resulting in an anti‐tumour effect. ATM is a kinase which responds to DNA double‐strand breaks (DSBs) while ATR recognizes single‐stranded breaks or areas of single‐stranded DNA (ssDNA). Multiple non‐DNA damaging stimuli, such as heat, chloroquine, and hypoxia, could increase the activity of ATM/ATR.^32^ The results also indicated that bortezomib treatment leads to the activation of ATM/ATR, inducing the glioma cells to undergo apoptosis, and inhibition of PLK4 expression enhances this effect. Given that the activation of ATM/ATR and the inhibition of mTOR are both closely related to hypoxia, we studied the expression of hypoxia and oxidative stress‐related molecules.

HIF‐α remains stable under normal oxygen conditions, while under hypoxia, HIF‐α is hydroxylated by specific prolyl hydroxylase domain‐containing proteins (PHDs) at two critical prolyl residues that results in its rapid degradation by the proteasome via ubiquitination.^33^ However, the relationship between ROS and HIF‐α remains obscure. A previous study found that hypoxia increases the production of ROS in the electron transport chain, resulting in increased stability and activity of HIF‐1α via inhibition of PHD activity,^34^ while another study found that increased production of ROS could promote the degradation of HIF‐1α via the ubiquitin‐proteasome system.^35^ Here, we found that the expression of HIF‐1α was decreased in the bortezomib treatment group, and therefore, we speculate that although hypoxia induces the production of ROS, which still did not resist the effect of hypoxia on expression of HIF‐1α, resulting in the decreased expression of HIF‐1α. Superoxide dismutase 1 (SOD1) catalyses the conversion of superoxide (O_2_^‐^) radicals into molecular oxygen (O_2_) or hydrogen peroxide (H_2_O_2_). Superoxide is the product of the oxygen metabolism process, and dysregulation of this process causes multiple types of cell damage.^36^ TRX has an important role in cellular and biological processes, including antioxidant defence, nitric oxide metabolism and control of cell death. Increased expression of TRX is critical for maintenance of the phenotypes and metastasis of tumours, as well as for promoting cell proliferation.^37^ TRX also participates in tumour development and metastasis via promotion of cell growth, resistance to apoptosis, and promotion of angiogenesis.^38^ In this study, we detected the expression of SOD1 and TRX in glioma cells and found that bortezomib treatment significantly decreased the expression of these two enzymes, inducing the oxidative stress process in glioma cells, leading to apoptosis of cells, and thus mediating an anti‐tumour effect. Besides, inhibition of PLK4 further enhances this effect. The BCL‐2 proteins are key regulators of the intrinsic apoptotic pathway, and among the members of the BCL‐2 family, Bcl‐2 mediates an anti‐apoptotic effect via binding and sequestering the monomeric activators or sensitizers of the apoptotic pathway (Bax or Bak).^39^ As a pro‐apoptotic effector, the activation of BCL‐2‐associated X protein (BAX) at mitochondrial surface results in mitochondrial outer membrane permeabilization (MOMP), causing the release of apoptotic proteins from the intermembrane space of mitochondrial. The down‐regulation of Bcl‐2 and up‐regulation of Bax following bortezomib treatment indicate that the apoptotic process was activated in the treated glioma cells, thus mediating an anti‐tumour effect, and inhibition of PLK4 further enhanced this effect. Matrix metalloproteinases (MMPs) are a family of extracellular zinc‐dependent endoproteinases that degrade ECM components.^40^ Among them, MMP‐2 and MMP‐9 are known to be closely associated with the development of cancer. Type IV collagen is the main component of the ECM and basement membrane, which form the first barrier for tumour metastasis. This barrier can be degraded by MMP‐2 after fibrillary collagen cleavage by collagenases.^41^ MMP‐9 is also a critical protease that has a vital role in multiple biological processes, especially in degradation of ECM through proteolytic cleavage to regulate ECM remodelling.^42^ MMP‐9 also plays an important role in regulation of tumour microenvironment via tumour invasion, angiogenesis and metastasis.^43^ In this study, we found that bortezomib treatment decreased the expression of MMPs, contributing to the maintenance of ECM and basement membrane, which inhibits the invasive and metastatic ability of cancer cells, and results in an anti‐tumour effect. Inhibition of PLK4 enhanced the effect of bortezomib in glioma cells. The MMPs are also regulated at the transcriptional level by multiple molecules, such as EGF, FGF, VEGF and TGF‐β.^44^ In this study, we found that the concentration of these cytokines decreased following bortezomib treatment, and this effect was further enhanced by inhibition of PLK4, resulting in the down‐regulation of the MMPs.

In this study, we established a cell model for PLK4 overexpression and knockdown in LN‐18, A172, and LN‐229 cells. We found that bortezomib treatment inhibited the proliferation of the glioma cells, and down‐regulation of PLK4 further enhanced this effect. The effect of bortezomib on glioma cells may be mediated by the PTEN/PI3K/AKT/mTOR signalling pathway, causing activation of the apoptotic pathway. We also found that bortezomib induced hypoxia and down‐regulated the MMPs, leading to a reconfiguration of the tumour microenvironment, resulting in inhibition of tumour cell proliferation. Studies in nude mice showed a similar trend as the in vitro experiments. And in order to verify these effects were not induced by PLK4 depletion or overexpression alone, we performed the key experiments on glioma cells without bortezomib treatment and found that there were no significantly changes in all experimental groups. Thus, we speculated that depletion of PLK4 could enhance the effect of bortezomib on glioma cells.

## CONFLICT OF INTEREST

None.

## AUTHOR CONTRIBUTION

Jing Wang, Dengpeng Ren and Yan Sun performed most of the experiments, analysed the data and wrote the manuscript. Chao Xu, Chunhong Wang and Rui Cheng performed the animal housing and sample collection. Lina Wang and Guijun Jia contributed to the sample collection. Jinrui Ren and Jiuhong Ma contributed to the data analysis. Yue Tu and Hongming Ji designed the research and revised the manuscript.

## Data Availability

Data of this manuscript are available from the corresponding author upon reasonable request.
